# 2146. Antimicrobial Activity of Aztreonam-avibactam, Ceftazidime-avibactam, and Meropenem-vaborbactam against Enterobacterales causing Bloodstream Infection in US Medical Centers (2020–2022)

**DOI:** 10.1093/ofid/ofad500.1769

**Published:** 2023-11-27

**Authors:** Helio S Sader, Cecilia G Carvalhaes, John H Kimbrough, Rodrigo E Mendes, Mariana Castanheira

**Affiliations:** JMI Laboratories, North Liberty, Iowa; JMI Laboratories, North Liberty, Iowa; JMI Laboratories, North Liberty, Iowa; JMI Laboratories, North Liberty, Iowa; JMI Laboratories, North Liberty, Iowa

## Abstract

**Background:**

As the frequency of Enterobacterales (ENT) producing metallo-β-lactamase (MBL) and/or OXA-48 is increasing in some US medical centers, effective antimicrobials to treat the infections caused by these organisms are urgently needed. Aztreonam-avibactam (ATM-AVI) is under clinical development for treatment of infections caused by Gram-negative bacteria, including MBL producers. We evaluated the activities of ATM-AVI, ceftazidime-avibactam (CAZ-AVI), meropenem-vaborbactam (MEM-VAB), and comparators against ENT isolated from patients with bloodstream infections (BSIs).

**Figure:**
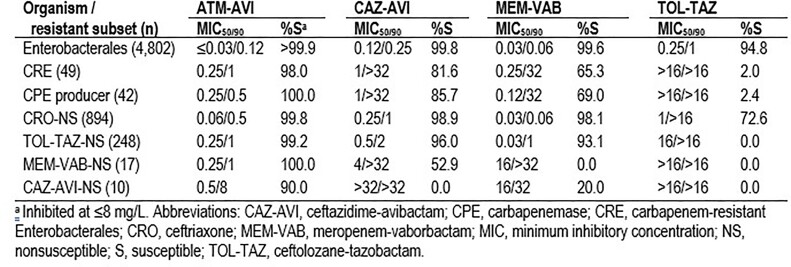

**Methods:**

4,802 ENT were consecutively collected (1/patient) from 72 US medical centers in 2020–2022 and susceptibility tested by CLSI broth microdilution method. ATM-AVI was tested with AVI at a fixed 4 mg/L. A pharmacokinetic/pharmacodynamic susceptible (S) breakpoint of ≤ 8 mg/L was applied for comparison. Carbapenem-resistant ENT (CRE) isolates were screened for carbapenemase (CPE) by whole genome sequencing.

**Results:**

ATM-AVI was highly active against ENT (Table); only 2 isolates showed ATM-AVI MICs > 8 mg/L: 1 MEM-S *E. coli* and 1 *E. aerogenes* (CRE). All CPE producers and 98.0% of CREs were inhibited at an ATM-AVI MIC of ≤ 8 mg/L. CAZ-AVI and MEM-VAB were active against 81.6% and 65.3% of CREs, respectively. Ceftolozane-tazobactam (TOL-TAZ) was active against only 72.6% of ceftriaxone (CRO)-nonsusceptible (NS) isolates. ATM-AVI retained activity (MIC, ≤8 mg/L) against all MEM-VAB-NS and 90.0% of CAZ-AVI-NS isolates. The most common CPEs were KPC-2/3 (57.1% of CREs), OXA-48–like (16.3%), and NDM (12.2%). A CPE gene was not observed in 14.3% of CREs. CAZ-AVI and MEM-VAB were active against 100.0% of KPC producers, but CAZ-AVI showed limited activity against MBL producers and MEM-VAB showed limited activity against OXA-48–like and MBL producers. The most active comparators against CRE were tigecycline (95.9%S), gentamicin (49.0%S), and amikacin (44.9%S).

**Conclusion:**

ATM-AVI demonstrated potent activity against a large collection ENT isolated from patients with BSI in US hospitals, including CREs and isolates resistant to recently approved β-lactamase inhibitor combinations. These results support further clinical development of ATM-AVI.

**Disclosures:**

**Helio S. Sader, MD, PhD, FIDSA**, AbbVie: Grant/Research Support|Basilea: Grant/Research Support|Cipla: Grant/Research Support|Paratek: Grant/Research Support|Pfizer: Grant/Research Support|Shionogi: Grant/Research Support **Cecilia G. Carvalhaes, MD, PhD**, AbbVie: Grant/Research Support|bioMerieux: Grant/Research Support|Cipla: Grant/Research Support|CorMedix: Grant/Research Support|Melinta: Grant/Research Support|Pfizer: Grant/Research Support **John H. Kimbrough, PhD**, AbbVie: Grant/Research Support|Basilea: Grant/Research Support|Pfizer: Grant/Research Support|Shionogi: Grant/Research Support **Rodrigo E. Mendes, PhD**, AbbVie: Grant/Research Support|Basilea: Grant/Research Support|Cipla: Grant/Research Support|Entasis: Grant/Research Support|GSK: Grant/Research Support|Paratek: Grant/Research Support|Pfizer: Grant/Research Support|Shionogi: Grant/Research Support **Mariana Castanheira, PhD**, AbbVie: Grant/Research Support|Basilea: Grant/Research Support|bioMerieux: Grant/Research Support|Cipla: Grant/Research Support|CorMedix: Grant/Research Support|Entasis: Grant/Research Support|Melinta: Grant/Research Support|Paratek: Grant/Research Support|Pfizer: Grant/Research Support|Shionogi: Grant/Research Support

